# Supplementary material for: Soil Fungal Community Characteristics at Timberlines of Sejila Mountain in Southeast Tibet, China
Source: J Fungi (Basel). 2023 May 21;9(5):596. doi: 10.3390/jof9050596 (PMC10219346; doi:10.3390/jof9050596)
Supplement: Supplementary file 1 [file jof-09-00596-s001.zip › jof-2352122-supplementary/Supplementary Materials/Supplementary figures.pdf]

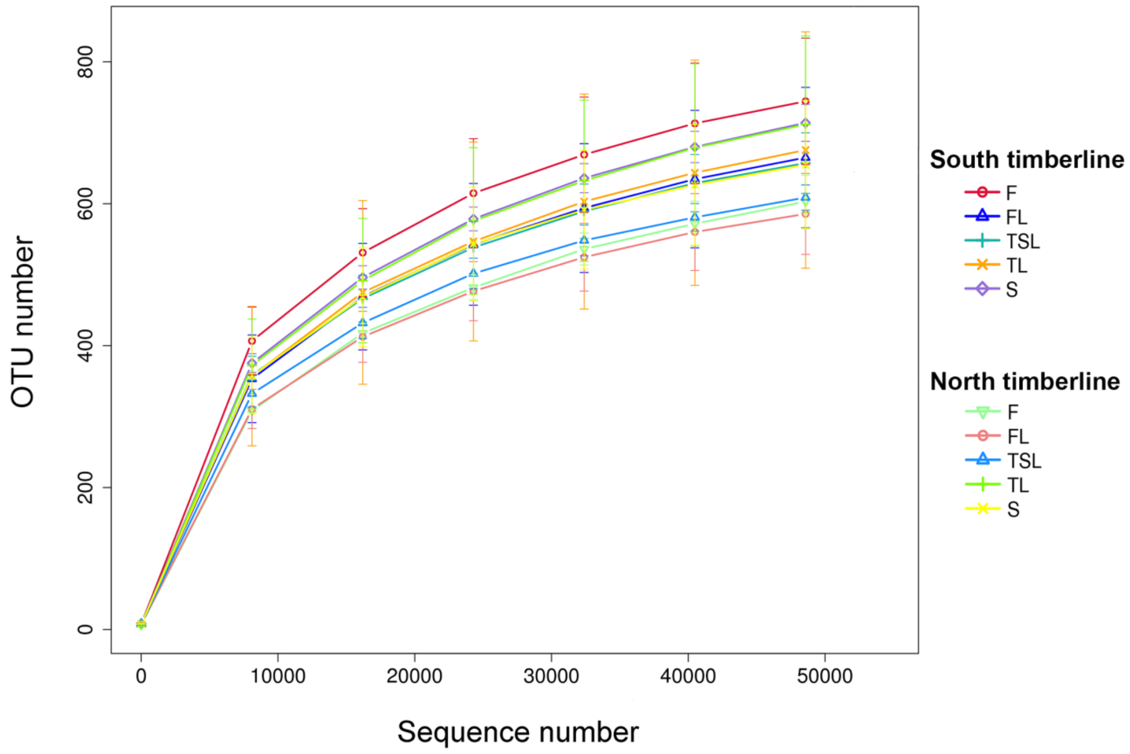

Figure S1. Rarefaction curves for soil fungal communities at the south and north timberlines of Sejila Mountain. F, forest; FL, forest line; TL, tree line; TSL, tree species line; S, shrub.

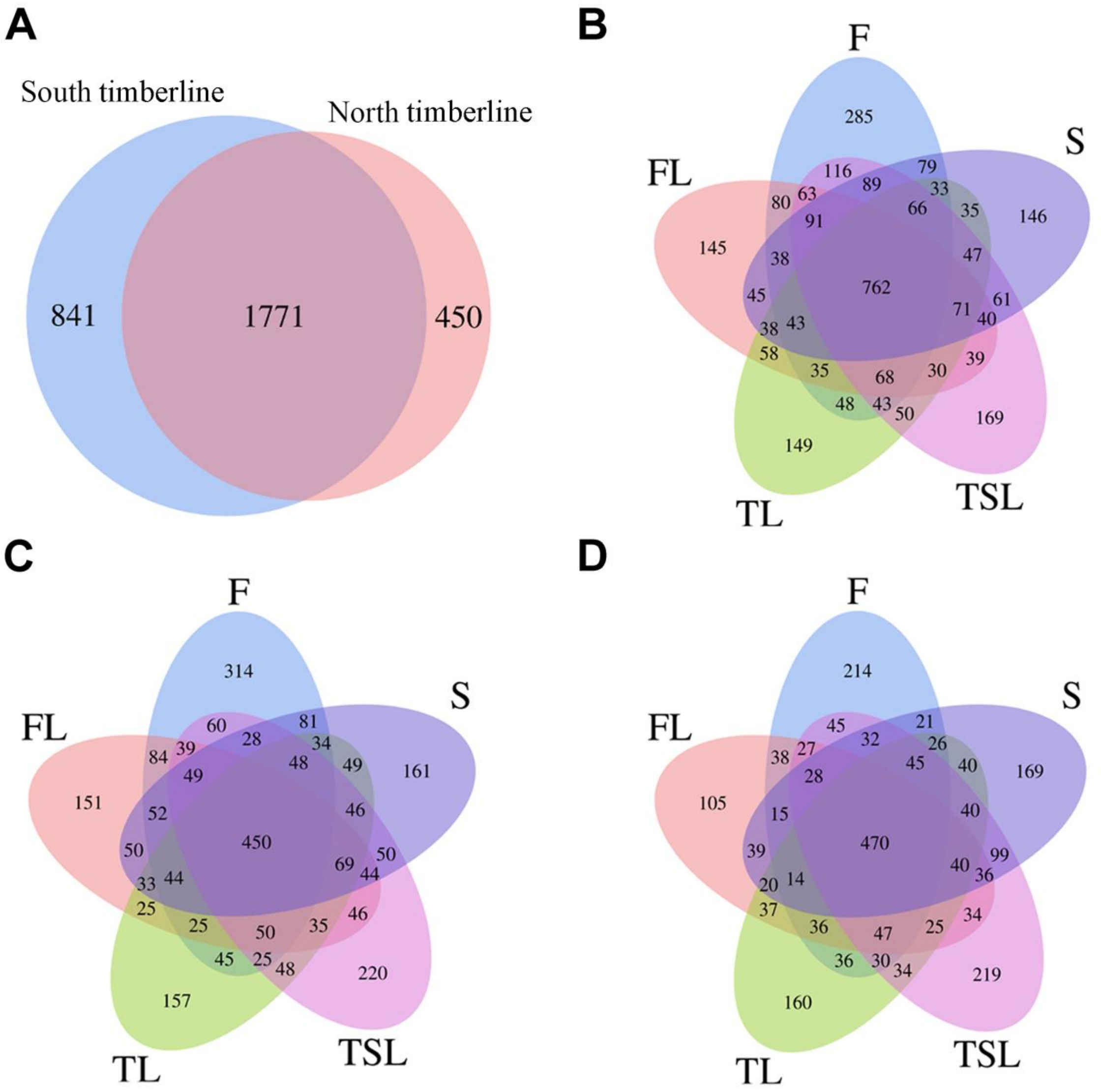

Figure S2. Venn diagrams for soil fungal OTUs at the south and north timberline of Sejila Mountain. **(A)** Two timberlines, **(B)** five vegetation zones, **(C)** south timberline, **(D)** north timberline. F, forest; FL, forest line; TL, tree line; TSL, tree species line; S, shrub.

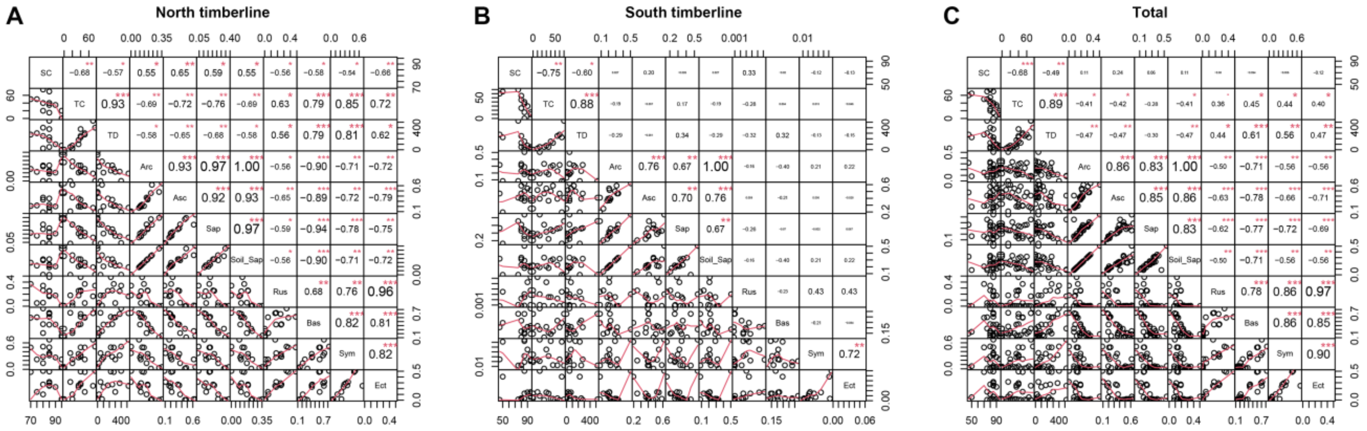

Figure S3. Pearson correlation between vegetation factors and main fungal genera, phyla, modes and guilds at the timberlines of Sejila Mountain. Correlations between vegetation factors and main fungal genera, phyla, modes and guilds at **(A)** the north timberline, **(B)** the south timberline and **(C)** the two timberlines. SC, shrub coverage; TC, tree coverage; TD, tree density; Arc, *Archaeorhizomyces*; Asc, Ascomycota; Sap, Saprotroph; Soil_Sap, Soil_Saprotroph; Rus, *Russula*; Bas, Basidiomycota; Sym, Symbiotroph; Ect, Ectomycorrhizal. *, **, *** indicate the significance levels of 0.05,
