# Supplementary material for: Soil Fungal Community Characteristics at Timberlines of Sejila Mountain in Southeast Tibet, China
Source: J Fungi (Basel). 2023 May 21;9(5):596. doi: 10.3390/jof9050596 (PMC10219346; doi:10.3390/jof9050596)
Supplement: Supplementary file 1 [file jof-09-00596-s001.zip › jof-2352122-supplementary/Supplementary Materials/Supplementary tables.pdf]

Table S1 Results for data preprocessing and quality control

Timberline Sites	Vegetation Zones	Plots	Raw PE	Raw Tags	Clean Tags	Effective Tags	Bases (nt)	Average Length (nt)	Q20	Q30	GC Content (%)	Effective Rate (%)
South	F	1	99387	97477	96988	67233	16381808	244	99	96	45	68
		2	98341	96219	95777	65931	14734545	223	99	97	45	67
		3	99159	97359	96746	66965	16425607	245	99	96	43	68
	FL	1	92819	90647	90173	67442	15581496	231	99	97	43	73
		2	83526	81904	81475	68173	14379488	211	99	97	45	82
		3	94602	93152	92930	63957	13445660	210	99	97	46	68
	TL	1	81068	78829	78553	60598	13491193	223	99	97	45	75
		2	91697	90053	89737	67083	15234191	227	99	97	43	73
		3	93379	92137	91965	66074	13122574	199	99	98	49	71
	TSL	1	91247	87955	87576	61114	13841887	226	99	97	45	67
		2	85547	83664	83384	65692	15306767	233	99	96	44	77
		3	98774	97224	96930	69292	14858314	214	99	97	45	70
	S	1	80456	77753	77439	69406	16066673	231	99	97	41	86
		2	91981	89366	89051	66090	15370038	233	99	96	43	72
		3	82237	79962	79404	68315	16639519	244	99	96	43	83
North	F	1	95226	93412	93085	62638	15188782	242	99	96	47	66
		2	85512	83636	82745	60264	15288927	254	99	96	46	70
		3	77454	76018	53339	49412	13958183	282	98	95	46	64
	FL	1	87833	85861	85233	62317	16473150	264	99	95	46	71
		2	97194	94246	93845	65282	16772547	257	99	96	45	67
		3	83056	80716	80150	66691	16080536	241	99	96	45	80
	TL	1	97007	95003	94117	69415	17494541	252	99	96	48	72
		2	87504	85026	84622	68183	15884753	233	99	96	46	78
		3	94375	91628	91104	65975	16212827	246	99	96	44	70
	TSL	1	90068	87495	60344	56911	15978084	281	98	95	46	63
		2	98957	97280	96995	68916	14728792	214	99	97	46	70
		3	99866	97535	97096	66756	15124275	227	99	97	44	67
	S	1	88652	85585	84937	62759	13502171	215	99	97	44	71
		2	91031	89372	89091	65579	15720696	240	99	96	44	72
		3	80804	78951	78499	68146	14582100	214	99	97	46	84
Total			2718759	2655465	2593330	1952609	457870124					
Average			90625	88516	86444	65087	15262337	235	99	96	45	72
Max			99866	97535	97096	69415	17494541	282	99	98	49	86
Min			77454	76018	53339	49412	13122574	199	98	95	41	63

[a] F, forest; FL, forest line; TL, tree line; TSL, tree species line; S, shrub; Raw PE, the raw PE reads produced by sequencer; Raw tags, the tags reads obtained by combining; Clean tags, the reads after filtered out raw reads with low quality and short length; Effective tags, the clean reads after filtered out chimeras; Bases, the base numbers of the effective reads; Average length, the average length of the effective reads; Q20 and Q30, the percentages of bases with quality values > 20 and 30 in the effective reads; GC content, the percentage of GC bases in the effective reads; Effective rate, the percentage of the effective reads in the raw PE reads.
